# Editorial: The role of parent-child interactions in developmental psychopathology: methodological and intervention challenges and opportunities

**DOI:** 10.3389/frcha.2024.1489804

**Published:** 2025-01-27

**Authors:** R. M. Pearson, B. Coetzee, T. J. Rochat

**Affiliations:** ^1^ School of Psychology, Faculty of Health and Education, Manchester Metropolitan University, Manchester, United Kingdom; ^2^ Centre for Academic Mental Health at the University of Bristol, Oakfield House, Bristol, United Kingdom; ^3^ UKMRC Integrative Epidemiology Unit, University of Bristol, Bristol, United Kingdom; ^4^ Department of Psychology, Faculty of Arts and Social Sciences, Stellenbosch University, Stellenbosch, South Africa; ^5^ SAMRC/Developmental Pathways to Health Research Unit, Faculty of Health Sciences, University of Witwatersrand, Johannesburg, South Africa

**Keywords:** parenting, mental health, interventions, development, method

**Editorial on the Research Topic**
The role of parent-child interactions in developmental psychopathology: methodological and intervention challenges and opportunities

Our research topic called on the field of developmental psychology to update the current status quo on the role of parent-child interactions in children's development. The field answered in robust and creative ways. That said, the collection still reflects historical challenges; an overrepresentation of research in early parenthood relative to the other developmental stages, the use of small observation studies, and a focus on mothers as compared to other caregivers was still apparent.

We summarize and discuss the edition and suggest next steps.

## Global representation

The diversity of submissions in the collection reflects a global interest in the topic; it also demonstrates the progress made in ensuring diverse populations are able to contribute knowledge to a field which has long been dominated by research from only a few ethnic groups in mostly high-income settings. The papers include studies from 17 countries representing both high-income and low- and middle-income contexts: Argentina, Belgium, Brazil, Canada, Finland, France, India, Israel, Italy, Japan, Kenya, Netherlands, Norway, Singapore, South Africa, the United Kingdom, and the United States of America.

## Developmental time periods

Most papers focused on infancy or early childhood and on interactions with mothers (12 out of 14), which reflects an imbalance in the broader understanding of the value of interaction and relationships across childhood and into adolescence and adulthood. This limits our understanding of the complex tapestry of relationship dynamics that emerge as children grow and, in particular, as their relational capacities develop with and through interactions with multiple family members including fathers (which only two papers focused on) and siblings. Children develop their capacities to contribute to interactions as they grow through developmental transitions which should develop their independence and autonomy. The current focus on the early years leaves several gaps in our understanding of how interaction develops alongside other socio-emotional and cognitive capacities, as well as how interactions shift and shape beyond the mother-child dyad. In addition, studies that map parenting to developmental growth and specific needs as and when they occur rather than time period would advance the field further.

This focus on the early years limits the potential to innovate in how we intervene at different developmental stages and contexts. It also introduces methodological vulnerabilities because the existing methodologies used for infancy research do not necessarily translate to older age groups. For example, concepts robust in infancy are taken as the gold standard, but we should not assume that concepts such as sensitivity and attachment present in the same way or have the same role in adolescence, a time in which successful developmental transitions would require greater autonomy and in which it may be adaptive to have “de-attach”. Studies included in the collections offer considerations of this and present potential methodological approaches that could inform observational research in middle childhood and adolescence (Wright et al., Lekhuleni et al.).

Much as looking forward to later development is important, considering pre-conception (including previous intergenerational pathways) and the prenatal period is key. While the pre-natal period is recognized biologically as having an impact on fetal and thus child development, its psycho-social influence on parent-child interactions is often overlooked. One study looked at the role of BMI in pre-pregnancy on later peer problems (Dow et al.), recognizing both biological and social pathways. No papers came forward looking at multi-generational influences or the role of new reproductive methods to conceive and the role of fertility treatment, which is a key change in contemporary pre-conception parenting journeys.

Parents are always parents, and their role and influence likely extend beyond the dependent years even into many generations after their death. Similarly, modern and complex ways of experiencing and contrasting the context of parent-child relationships were not extensively explored in this collection; for example, no studies captured the complexities of half, step, adoptive, and single parents or same-sex parenthood.

## Methodological advances

We specified that we were interested in interactions, and this was considered by authors in many ways. Some studies examined interactions in their simplest forms, exploring well known constructs like “responsiveness”, while others explored more complex coding matrices on mutual affect and what can be learned from micro-coding, emotional recognition, and the use of Artificial Intelligence (AI) and how these technologies may support an openness to new understandings not limited by historical knowledge and standards. In addition, more global assessments of interactions were included, such as concepts of sensitivity or attachment that re not taken from one moment alone. Rather, concepts such as sensitivity are taken as greater than the component micro moments and, in some ways, rely on a subjective sense by the observer. Greater periods of time were generally assessed by self-reports of perceptions; these included simple proxies of interactions based on working hours in Japan, the use of routines during lockdown, or the recall of childhood events such as neglect or emotional trauma in parent-child relationships.

The collection does suggest researchers are beginning to grapple with this and expand methodologies to include bio-psycho-social processes as a means to engage with the potential multiple and competing pathways that may be involved in outcomes (see for example Braithwaite's examination of interactions and their biological signals embedded in DNA methylation). These different ways of “knowing” or gaining insights into the parent-child interaction or relationship were exciting to see and the next steps may include triangulation and understanding.

Other exciting methodological developments presented as part of the collection include the richness of new tasks and protocols. These explored creative approaches in protocol development to observe parent-child interactions, such as using free play, structure, attachment protocols, and specific games in research “labs” or at home (1). These also extend to the use of new technology such as wearable cameras and AI, which offer exciting opportunities for future research. Similarly, a refreshing theme of co-production and creativity in engagement emerged from the papers as a new norm and foundation to challenge ideas and develop methodology. Such co-production included art (Culpin et al.), mobile pop-up events (Wright et al.), and new ways of engaging in “talk” with adolescents in groups (Lekhuleni et al.).

In addition, to gain statistical power to reach a larger sample size, it is key that more work is done in collaboration and that data sharing and harmonization is *a priori*ty. Some important examples are found in this collection, with studies designing aligned protocols in multiple settings (Bornstein et al.) or protocols being developed to harmonize data from consortiums of cohort studies that followed different protocols and did not initially aim to be brought together (e.g., Dream Big Consortium) (2).

## Intervention advances

Only one study investigated the role of interventions (Braithwaite et al.). However, many more provided evidence.

For example, the use of wearable cameras to enhance video-feedback via first person perspective was noted in three of the included papers in this topic. This is important as a recent meta-analysis of 59 randomized controlled studies found that psycho-educational advice alone is not effective in reducing later mental health disorders (3). Rather, relational support at home, in practice, is the key causal ingredient in parenting interventions in preventing mental health risk and increasing parents' self-efficacy.

Wearable camera devices for both children and babies are described in this topic (Skinner et al.); as they capture the same moment for both parties, the devices enable the viewing of shared emotions and how the interaction was different from different viewpoints, aiding introspection and reflective functioning. The co-production development work highlighted in this topic (Wright et al.) reports that parents notice more mental state signals when viewing first-person perspective footage and can more easily re-live the moment when it is viewed back “through their eyes”.

In addition, some of the AI discussed in this issue, such as face-reader, could support interventions. Finding moments of strength is key to video-feedback but is limited by practitioners scanning through lots of footage and hampered by natural human subjectivity/error. Face-reader technology, described in this topic, records intensity of emotions (such as sadness or happiness) concurrently in parent and child on a scale between 0 and 1, every 0.02 s, allowing statistical modelling of subtle transitions in emotion. Face-reader processing systems could automatically indicate key moments, drastically saving time and potentially standardizing the selection of footage alongside personalized input, where AI and therapists work together.

## Interventions in context

Finally, studies in this collection looked at the role of previous contexts on later outcomes (Hill et al.), demonstrating that when trauma happens (in this case exposure to violence) the child's attachment history is relevant. This is also seen in trauma-informed care. How trauma may interact with mindfulness to predict later eating disorders was also explored (Royer et al.). In combination, these papers support more personalized and systemic approaches to parenting interventions, considering a developmental lens.
